# Acupuncture modulates stress response by the mTOR signaling pathway in a rat post-traumatic stress disorder model

**DOI:** 10.1038/s41598-018-30337-5

**Published:** 2018-08-08

**Authors:** Ju-Young Oh, Yu-Kang Kim, Seung-Nam Kim, Bombi Lee, Jae-Hwan Jang, Sunoh Kwon, Hi-Joon Park

**Affiliations:** 1 Acupuncture and Meridian Science Research Center, Kyung Hee University, 26 Kyungheedae-ro, Dongdaemun-gu, Seoul, 02447 Republic of Korea; 2 Department of Korean Medical Science, Graduate School of Korean Medicine, Kyung Hee University, 26 Kyungheedae-ro, Dongdaemoon-gu, Seoul, 02447 Republic of Korea; 3 BK21 PLUS Korean Medicine Science Center, College of Korean Medicine, Kyung Hee University, 26 Kyungheedae-ro, Dongdaemoon-gu, Seoul, 02447 Republic of Korea; 4 College of Korean Medicine, Dongguk University, 32, Dongguk-ro, Ilsandong-gu, Goyang-si, Gyeonggi-do 10326 Republic of Korea; 5 Korean Institute of Oriental Medicine, 1672 Yuseong-daero, Yuseong-gu, Daejeon, 34054 Republic of Korea

## Abstract

Post-traumatic stress disorder (PTSD) is a psychiatric disease that can form following exposure to a traumatic event. Acupuncture has been proposed as a beneficial treatment for PTSD, but the underlying mechanisms remain unclear. The present study investigated whether acupuncture improves depression- and anxiety-like behaviors induced using a single prolonged stress (SPS) as a PTSD rat model. In addition, we investigated whether the effects were mediated by increased mTOR activity and its downstream signaling components, which contribute to protein synthesis required for synaptic plasticity in the hippocampus. We found that acupuncture at HT8 significantly alleviated both depression- and anxiety-like behaviors induced by SPS in rats, as assessed by the forced swimming, elevated plus maze, and open field tests; this alleviation was blocked by rapamycin. The effects of acupuncture were equivalent to those exerted by fluoxetine. Acupuncture regulated protein translation in the mTOR signaling pathway and enhanced the activation of synaptic proteins, PSD95, Syn1, and GluR1 in the hippocampus. These results suggest that acupuncture exerts antidepressant and anxiolytic effects on PTSD-related symptoms by increasing protein synthesis required for synaptic plasticity via the mTOR pathway in the hippocampus. Acupuncture may be a promising treatment for patients with PTSD and play a role as an alternative PTSD treatment.

## Introduction

Post-traumatic stress disorder (PTSD) is a debilitating mental disorder that can follow exposure to traumatic events, such as actual or threat of death, severe injury, or sexual violation. The Diagnostic and Statistical Manual of Mental Disorder 5th edition (DSM-V) defines PTSD according to four distinct diagnostic symptom clusters: recurring memories of the traumatic event, avoidance, negative alterations in cognitions and mood, and alterations in arousal and reactivity^1,2^. PTSD is often comorbid with depression and other anxiety disorders^3^. Psychotherapy and medication, the typical treatment methods for patients with PTSD, involve many years of personal and group therapy and the administration of drugs, including antidepressants and anxiolytic drugs. Research has found significant individual differences in the response to such therapies, as well as side effects^4,5^; therefore, better therapies are needed to treat PTSD.

Acupuncture, a treatment with few side effects, is a major therapy that has been used in East Asia for thousands of years to treat various medical and psychiatric conditions^6^. Several studies have substantiated its benefits for depression and anxiety^7–10^. Acupuncture administered at point HT7 has been found to relieve emotional problems, especially excessive anxiety and worry^11^, and acupuncture at point HT8 has been reported to have therapeutic effects on mental disorders^12^. Possible therapeutic mechanisms of acupuncture include regulation of neurotransmitter levels^13^, the neuroendocrine system^14^, and inflammatory cytokines^15^. However, the therapeutic mechanism of acupuncture associated with the effects has yet to be investigated in PTSD animal model.

In general, mental stress increases sympathetic activity. Acupuncture is known to stabilize the central nervous system (CNS) and control the HPA axis to treat stress-induced diseases. In addition, acupuncture is used for depression and anxiety, which are the most frequent complaints of psychiatric complaints in Complementary Alternative Medicine (CAM) clinic^16–21^. Acupoint HT8 has been used to sedate “*Fire qi*” according to *Sa-Am* acupuncture theory of Korean Medicine, with our interpretation of reducing depression or anxiety. Kim *et al*. reported that acupuncture at HT8 normalized the mental stress-evoked alteration of autonomic nerve system^22^.

PTSD animal models include many stress paradigms such as predator/predator-odor stress, exposure to inescapable electric shocks, and the single prolonged stress (SPS) paradigm^23–25^. In the SPS rat model, animals that were treated with predictable continuous trauma showed depression- and anxiety-like behaviors, which are psychological symptoms of PTSD^26,27^. Therefore, this animal model offers advantages for studying the behavioral and biological mechanisms of PTSD.

The mammalian target of rapamycin (mTOR), a large serine/threonine kinase, regulates the initiation of protein translation in the body. mTOR acts both as a node of convergence downstream of the receptors and a regulator of several signaling pathways^28^. Activation of mTOR contributes to the phosphorylation of two major substrates downstream, ribosomal protein S6 kinase (p70S6K) and translation repressor protein 4E-binding protein 1 (4E-BP-1); activation of these control elements induces protein translation. Dysregulation of mTOR is implicated in the etiology of various psychiatric disorders, including depression^29^. Previous studies showed a loss in mTOR signaling in the hippocampus of subjects with depression and anxiety disorders^22,23^. Several animal studies have indicated that exposure to chronic unpredictable mild stress (CUMS) elicits deficits in mTOR signaling pathway components in the hippocampus^24,25^. Furthermore, pretreatment with the mTOR inhibitor rapamycin abolishes this antidepressant effect in a PTSD rat model^30^. These results indicate the indispensable role of mTOR in mediating antidepressant effects. Based on these findings, we hypothesized that the antidepressant-like effect of acupuncture may occur via activation of the upstream and downstream targets of mTOR signaling.

Stress is thought to induce changes in nerve cells and impair the number and function of synapses, leading to depression^31,32^. Damaged synapses are usually accompanied by loss of synaptic proteins important for synaptic functions, such as post-synaptic density 95 (PSD95) and the pre-synaptic protein synapsin I (Syn1)^33,34^. PSD95, the main protein of excitatory postsynaptic density material, and Syn1, widely distributed in the presynaptic vesicle membrane, play important roles in synaptic plasticity^35^. mTOR regulates cell growth, proliferation, and synaptic plasticity by activating protein synthesis^26,27^. Moreover, marked deficits in synaptic proteins are associated with dysregulation of mTOR signaling in depressed subjects^36^. Activation of mTOR is associated with local protein synthesis in the synapse, promoting the expression of synaptic connection proteins such as PSD95, Syn1, and glutamate receptor 1 (GluR1)^37^. Considering the important role of the mTOR signaling pathway in the regulation of protein translation and synaptic plasticity, novel drugs that regulate mTOR signaling activity, followed by enhanced mTOR-dependent protein synthesis, could be useful for producing antidepressant effects. Therefore, we hypothesized that acupuncture may exert antidepressant effects by activating the mTOR signaling pathway, reversing synaptic protein loss and impaired function and improving synaptic plasticity.

This study investigated whether acupuncture has beneficial effects on the depression- and anxiety-like behaviors in the SPS rat model of PTSD using the forced swimming test (FST), open field test (OFT), and elevated plus maze (EPM) test. The effects of acupuncture on the modulation of mTOR signaling pathway in the hippocampus were measured. Changes in the synaptic proteins associated with synaptic plasticity were also assessed.

## Results

### Selection of the most effective acupoint for treating the SPS induced PTSD

**Figure 1:**
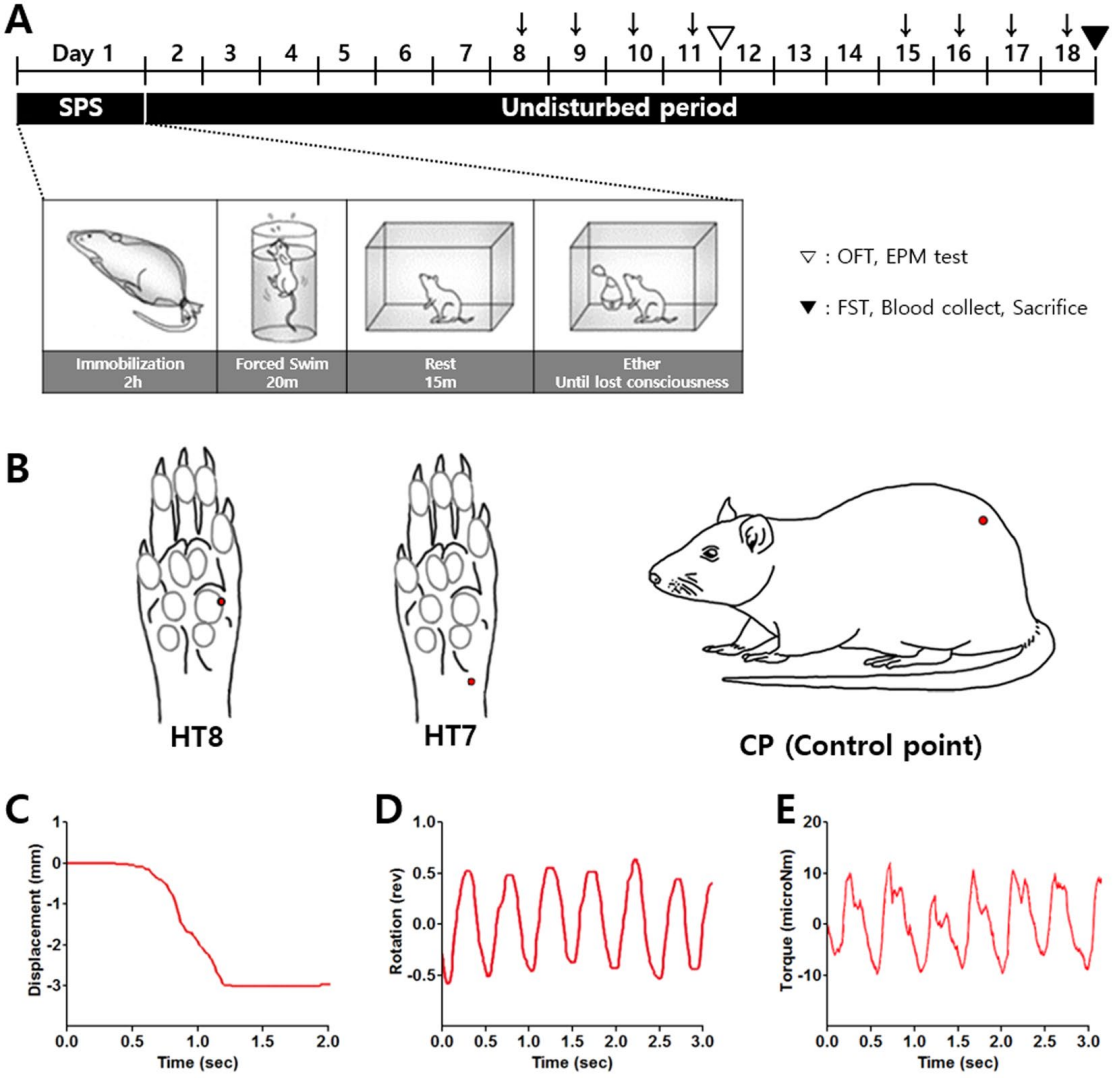
Experimental schedules of developing SPS and details of acupuncture needling. SPS-induced depression- and anxiety- like behaviors, and acupuncture stimulation in the rats. (**A**) OFT and EPM test were tested on day 11 (▽). FST, blood collect and sacrificed were conducted on day 18 (▼). (↓Day of acupuncture, fluoxetine or rapamycin injection) (**B**) Locations of the acupuncture treatment (red circle) HT8 ( *Sobu*), HT7 ( *Shenmen*) and control point (CP). HT8 is located on the palm of the fore paw, in the depression between the fourth and fifth metacarpal bones, proximal to the fifth metacarpophalangeal joint and HT7 is located on the anteromedial aspect of the wrist, radial to the flexor carpi ulnaris tendon, on the palmar wrist crease. CP refers to control point. (**C**–**E**) ACUSENSOR measurements for acupuncture stimulation at HT8. The depth of needling (displacement, **C**) and the turning of the needle (rotation, **D**) were measured using a motion sensor. The rotational force acting on the needle (torque, **E**) was measured using a force sensor. The needle rotated at a rate of 2 spins per second for 30 seconds. SPS, Single prolonged stress; OFT, Open field test; EPM, Elevated plus maze; FST, Forced swimming test.

To determine the most effective acupoint for treating PTSD, we first compared the antidepressant and anxiolytic effects of acupuncture at three points, namely HT8, HT7, and a control point (CP), in SPS rats using the FST, OFT, and EPM (Fig. 1).

**Figure 2:**
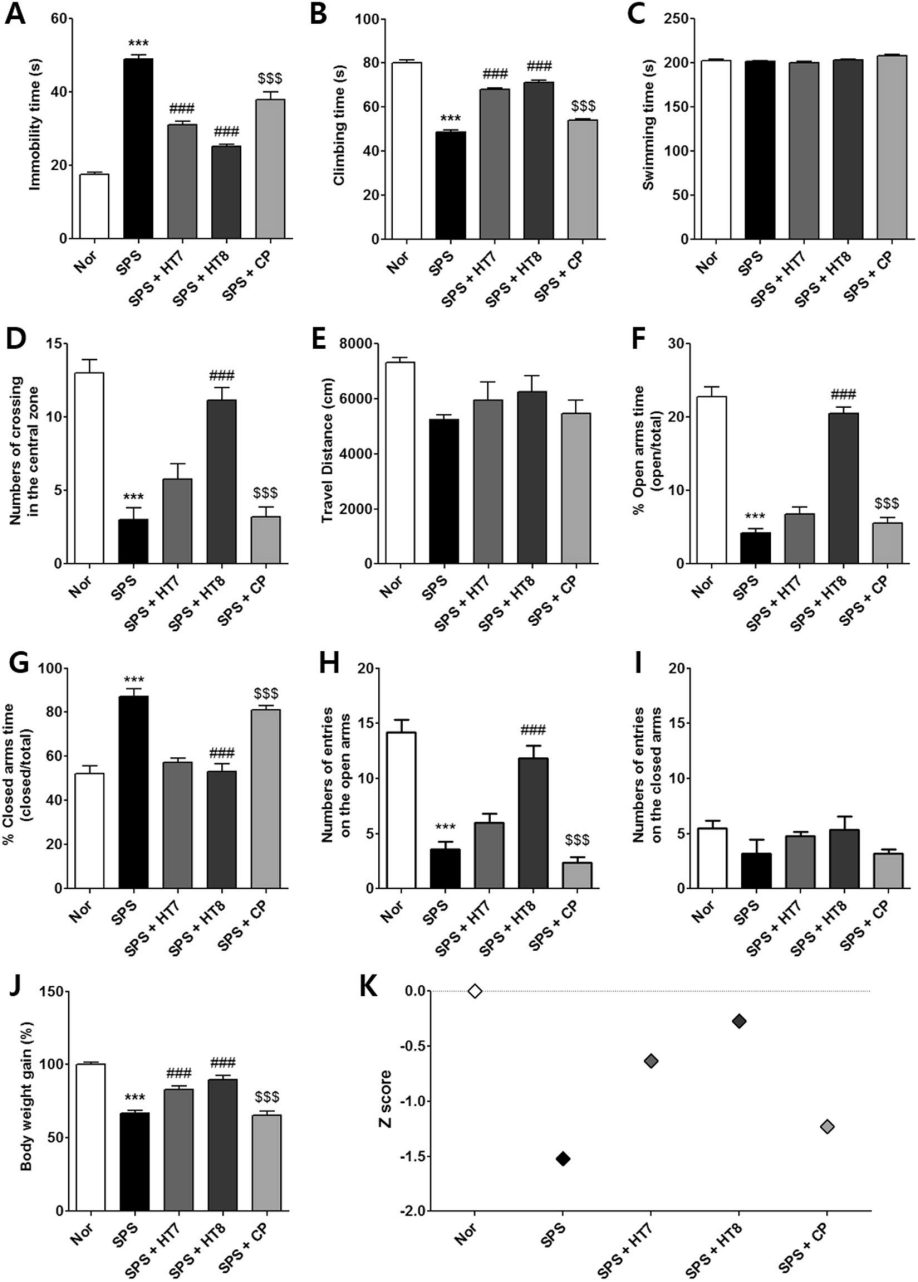
Effects of acupuncture at HT8 on depression- and anxiety-like behaviors and, blood levels of corticosterone and CRF expression in the PVN of the SPS model of PTSD. (**A**) Variations in body weight of rats during 18days. HT8 group and Flu group has significantly induced the weight loss in rats as from the 9 day vs. SPS group. (↓Day of acupuncture stimulation or fluoxetine injection.) Effects of HT8 on depression- and anxiety-like effects in (**B**–**D**) FST, (**E**–**G**) OFT and (**H**–**K**) EPM test in SPS model of PTSD. (**L**) The concentration of corticosterone in the serum was detected by ELISA. (**M**,**N**) CRF expression levels in the PVN of hypothalamus by immunofluorescence. ***p < 0.001, **p < 0.01 vs. Nor group, ^###^*p* < 0.001, ^##^*p* < 0.01, ^#^*p* < 0.05 vs. SPS group, ^$$$^*p* < 0.001, ^$$^*p* < 0.01 vs. HT8 group. n = 5–6 each. Scale bar: 250 μm. Nor, normal; SPS, single prolonged stress; HT8, acupuncture group with the HT8; CP, control point acupunctured group; Flu, fluoxetine; FST, forced swim test; OFT, open field test; EPM, elevated plus maze.

The rats’ FST performance was analyzed by one-way analysis of variance (ANOVA). The results showed that acupuncture treatments at the HT7 (SPS + HT7) and HT8 (SPS + HT8) had a significant effect on immobility [F_4,31_ = 105.5, *p* < 0.0001] and climbing time [F_4,31_ = 185.4, *p* < 0.0001]; however, swimming time was not significantly affected [F_4,31_ = 3.575, *p* = 0.0183]. *Post hoc* analyses showed that rats in the SPS group exhibited more immobility during FST than did normal untreated rats (Nor group) (*p* < 0.001, Fig. 2A). However, rats in the SPS + HT7 and SPS + HT8 groups displayed significant decreases in the duration of immobility compared to the SPS group (each *p* < 0.001). Analysis of climbing time showed that rats in the SPS group exhibited a significant decrease in climbing time during the FST compared to the Nor group (*p* < 0.001, Fig. 2B). In contrast, both SPS + HT7 and SPS + HT8 groups exhibited significant increases in climbing time (each *p* < 0.001; vs. SPS), whereas the SPS + CP group showed no change. However, there were no significant differences in swimming times among the SPS groups (Fig. 2C).

One-way ANOVA of performance on the OFT revealed significant effects of acupuncture on the frequency of line crossing [F_4,26_ = 28.59, *p* < 0.0001]. *Post hoc* analyses showed a significant increase in the number of line crossings in the SPS + HT8 group compared with the SPS group (*p* < 0.001). In contrast, the SPS + HT7 and SPS + CP groups showed no significant improvements (Fig. 2D). Travel distance did not differ among the groups [F_4,23_ = 2.188, *p* = 0.1093], indicating that their motor functioning did not differ significantly (Fig. 2E).

One-way ANOVA of EPM performance showed that the percentage of time spent in the open [F_4,26_ = 85.98, *p* < 0.0001] (Fig. 2F) and closed [F_4,24_ = 31.37, *p* < 0.0001] (Fig. 2G) arms of the maze differed significantly among the five groups. *Post hoc* comparisons revealed a significant decrease in the percentage of time spent in the open arms of the maze among the SPS rats compared to the Nor group (*p* < 0.001, Fig. 2F). However, the SPS + HT8 group showed a significant restoration of time spent in the open arms of the maze compared to that spent by the SPS group (*p* < 0.001). Statistical analyses of EPM behavior showed that the number of entries into the open arms of the maze differed significantly among the five groups [F_4,26_ = 28.59, *p* < 0.0001]. *Post hoc* comparisons revealed a significant decrease in the number of entries into the open arms in the SPS group compared to the Nor group (*p* < 0.001, Fig. 2H). However, rats in the SPS + HT8 group exhibited a significant restoration in the number of entries into the open arms compared to the SPS group (*p* < 0.001). As there were no significant differences in the number of closed arm entries among groups in the EPM test [F_4,26_ = 1.589, *p* = 0.2126] (Fig. 2I), the observed anxiety-like behaviors of the SPS-induced rats are likely not attributable to differences in their locomotor activities. The increased number of entries by the SPS + HT8 group into the open arms was almost comparable to that of the Nor group.

We measured the body weight of each rat in the various groups for 18 days. Analysis of the body weight values revealed a significant gradual reduction in body weight gain in the SPS group relative to the Nor group [F_4,24_ = 38.02, *p* < 0.0001]. During the same period, the SPS + HT7 and SPS + HT8 groups showed slight alleviation of those reductions in body weight gain (*p* < 0.001) (Fig. 2J). In all of the experiments, acupuncture treatment at the control point (SPS + CP) showed no positive effects.

PTSD has diverse symptoms including depression and anxiety; therefore, this animal model was tested for two different behaviors simultaneously. To clarify the effect of the treatment, we analyzed data for both behaviors together by normalizing the results to those of the Nor group and expressing the data as *Z*-scores. Marked impairment was observed in the SPS group (*Z* = −1.52) compared with the Nor group (*Z* = 0). SPS + HT8 treatment (*Z* = −0.27) yielded a significant improvement compared to the SPS group. However, only slight improvement was observed in the SPS + HT7 (*Z* = −0.63) and SPS + CP (*Z* = −1.23) groups (Fig. 2K).

### Effects of acupuncture at HT8 on depression- and anxiety-like behaviors in SPS-induced PTSD rats

**Figure 3:**
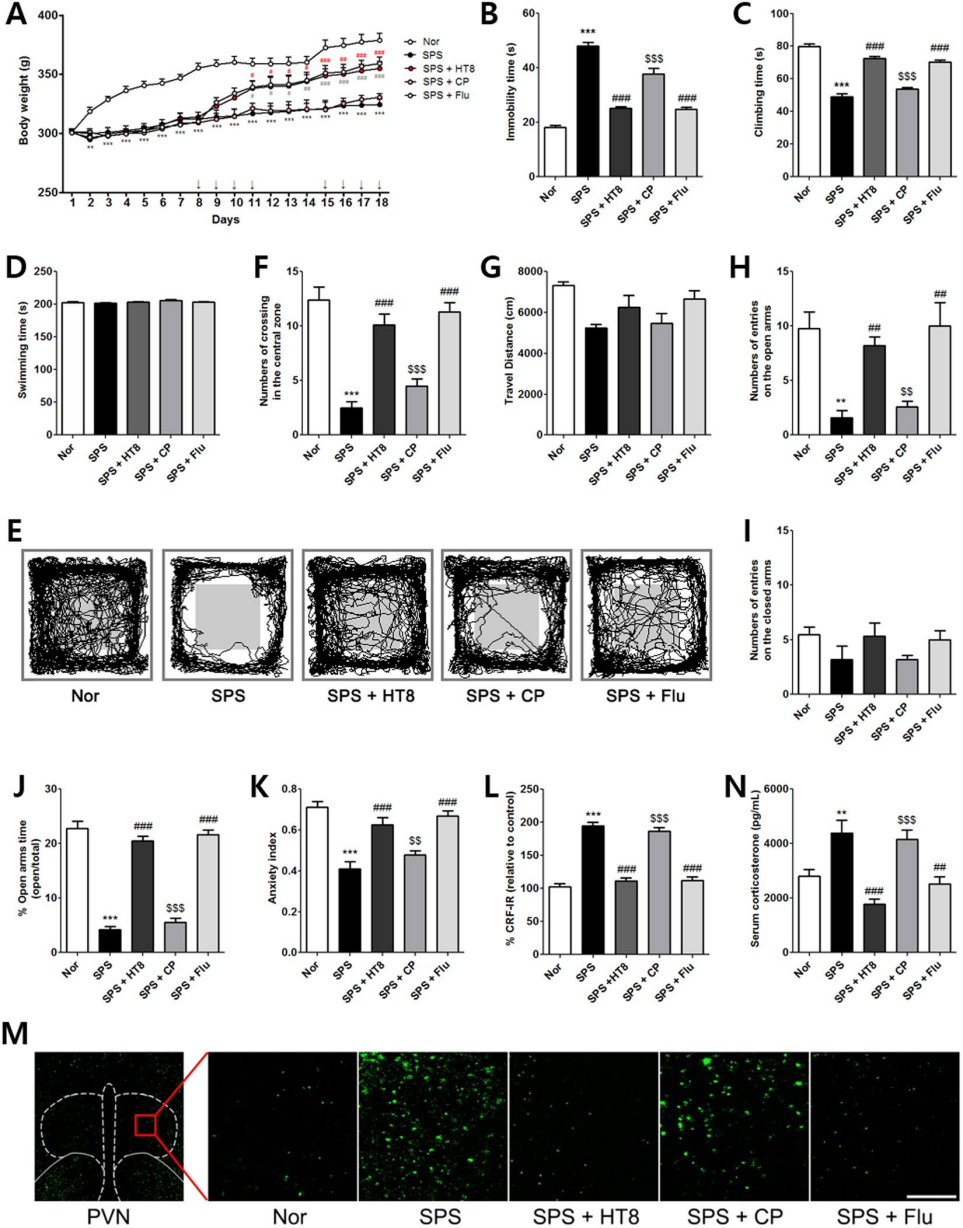
Effects of acupuncture at HT8 on depression- and anxiety-like behaviors and CRF expression in the PVN, and blood levels of corticosterone of the SPS model of PTSD. (**A**) Variations in body weight of rats during 18days. HT8 group and Flu group has significantly induced the weight loss in rats as from the 9 day vs. SPS group. (↓Day of acupuncture stimulation or fluoxetine injection.) Effects of HT8 on depression- and anxiety-like effects in (**B**–**D**) FST, (**E**–**G**) OFT and (**H**–**K**) EPM test in SPS model of PTSD. Acupuncture at HT8 modulated the HPA axis. (**L**–**M**) CRF expression levels in the PVN of hypothalamus by immunofluorescense. (**N**) The concentration of corticosterone in the serum was detected by ELISA. ***p < 0.001, **p < 0.01 vs. Nor group, ^###^*p* < 0.001, ^##^*p* < 0.01, ^#^*p* < 0.05 vs. SPS group, ^$$$^*p* < 0.001, ^$$^*p* < 0.01 vs. HT8 group. n = 5–6 each. Scale bar: 250 μm. Nor, normal; SPS, single prolonged stress; HT8, acupuncture group with the HT8; CP, control point acupunctured group; Flu, fluoxetine; FST, forced swim test; OFT, open field test; EPM, elevated plus maze.

The rats were weighed over the course of SPS induction and treatments for 18 days. Two-way ANOVA showed a significant difference among the groups in weight change [F_4,27_ = 32.70, *p* < 0.0001] and in the timing of the effect (days) [F_17,27_ = 40.64, *p* < 0.0001]. On *post hoc* testing, the SPS group showed a significant decrease in body weight compared to the Nor group from day 2 of SPS induction (*p* < 0.01). However, after day 4 of SPS induction, rats in the SPS + HT8 group and in the SPS group treated with fluoxetine (SPS + Flu) gained a significant amount of body weight compared to the SPS-induced group. During the same period, the SPS + CP group showed no body weight recovery (Fig. 3A).

One-way ANOVA of performance on the FST indicated significant group effects on immobility [F_4,32_ = 104.7, *p* < 0.0001] and climbing time [F_4,32_ = 79.22, *p* < 0.0001], but not on swimming time [F_4,32_ = 1.091, *p* = 0.3801]. *Post hoc* analyses showed a significant depression-like phenotype in the SPS group, namely increased duration of immobility, compared to the Nor group (*p* < 0.001, Fig. 3B). In contrast, the SPS + HT8 group exhibited a marked decrease in the duration of immobility compared to the SPS group (*p* < 0.001), indicating that acupuncture treatment at HT8 alleviated depression-like behaviors. Analysis of climbing time, another key indicator of depressive behavior, revealed that the SPS group exhibited significantly decreased climbing time compared to the Nor group (*p* < 0.001); however, the SPS + HT8 group showed recovery of climbing time (*p* < 0.001; vs. SPS, Fig. 3C). The SPS-induced groups displayed little variation in swimming time, indicating that the above results are caused by depression-like behavior, not by motor function deficits (Fig. 3D).

The OFT was used to study anxiety-like and exploratory behavior. Rats were exposed to the novel environment in the open field apparatus, and the routes traveled were measured. One-way ANOVA of OFT data revealed a significant group effect [F_4,49_ = 24.73, *p* < 0.0001]. *Post hoc* analysis showed that the total number of line crossings was reduced significantly in the SPS group (*p* < 0.001; vs. Nor, Fig. 3E,F). However, those treated with acupuncture at HT8 showed significant recovery of the total number of line crossings (*p* < 0.001, Fig. 3E,F), indicating lower anxiety. However, the travel distance did not differ among the groups [F_4,28_ = 2.779, *p* = 0.0498], indicating that their motor function did not differ significantly (Fig. 3G).

To observe the anxiety-like behaviors, we assessed the rats during open arm exploration in the EPM. One-way ANOVA revealed significant group effects on the number of entries into the open arms [F_4,24_ = 9.944, *p* = 0.0001] but in the closed arms [F_4,24_ = 1.459, *p* = 0.2470]. *Post hoc* comparisons showed that the SPS group spent significantly less number of crossing in the open arms compared to the Nor group (*p* < 0.01), indicating that SPS induced anxiety-like behavior (Fig. 3H). In contrast, a significant restoration in the number of entries into the open arms was observed in the SPS + HT8 group when compared to the SPS group (*p* < 0.01). Such change was not found in the CP-treated group. There were no significant differences in the number of closed arm entries between SPS and SPS + HT8 groups, suggesting that locomotor function did not differ among the groups (Fig. 3I). One-way ANOVA revealed a significant group effect in time spend on EPM performance [F_4,33_ = 76.94, *p* = 0.2763]. *Post hoc* analysis showed that the SPS group spent a significantly lower percentage of time in the open arms of the maze compared to the Nor group (*p* < 0.001, Fig. 3J). Overall, one-way ANOVA revealed a significant group effect [F_4,33_ = 15.81, *p* < 0.0001] in the anxiety index calculated based on the number of visits to and time spent in the open and closed arms. The anxiety index was higher in the SPS + HT8 group compared to the SPS group (*p* < 0.001, Fig. 3K), indicating anxiolytic effects.

### Effects of acupuncture at HT8 on serum corticosterone levels and corticotropin-releasing factor (CRF) expression in the paraventricular nucleus (PVN) of the hypothalamus following SPS

We examined the levels of serum corticosterone and changes in CRF-expressing neurons in the PVN of the hypothalamus. Serum corticosterone and hypothalamic CRF were used as indices of hypothalamic–pituitary–adrenal (HPA) axis activity.

Analysis of serum corticosterone levels revealed significant differences among the groups [F_4,28_ = 82.36, *p* < 0.0001]. As shown in Fig. 3L, *post hoc* comparisons showed a significantly increased level of serum corticosterone in the SPS group compared to the Nor group (*p* < 0.01). In contrast, the SPS + HT8 and SPS + Flu groups evidenced a significant decrease in the concentration of corticosterone compared to that in the SPS group (*p* < 0.001). These data suggest that acupuncture at HT8 inhibited HPA axis hyperactivity in the SPS model of PTSD.

Analysis of the CRF expression levels in the PVN revealed significant differences among the groups [F_4,26_ = 68.52, *p* = 0.9993]. *Post hoc* comparisons revealed that the SPS group showed higher levels of CRF expression than did the Nor group (*p* < 0.001). However, the SPS + HT8 and SPS + Flu group showed a lower level of CRF expression significantly than the SPS group did (*p* < 0.001). The CP-treated group did not show this recovery compared to the SPS group (Fig. 2M,N).

### Effects of acupuncture at HT8 on the mTOR signaling pathway in the hippocampus following SPS

To investigate whether the antidepressant and anxiolytic effects of acupuncture are associated with the mTOR signaling pathway, we analyzed the protein expression levels of key proteins in this pathway. The proteins assessed were p-extracellular signal-regulated kinase (ERK) forms 1 and 2, p-Akt, p-mTOR, p-p70S6K, p-4E-BP-1, and p-CREB. One-way ANOVA revealed significant differences in protein expressions of p-ERK/ERK [F_4,10_ = 10.95, *p* = 0.0011], p-Akt/Akt [F_4,10_ = 43.05, *p* < 0.0001], p-mTOR/mTOR [F_4,10_ = 24.43, *p* < 0.0001], p-p70S6K/β-actin [F_4,10_ = 37.26, *p* < 0.0001], p-4E-BP-1/β-actin [F_4,10_ = 15.74, *p* = 0.0003], and p-CREB/β-actin [F_4,10_ = 164.8, *p* < 0.0001] in the hippocampus.

**Figure 4:**
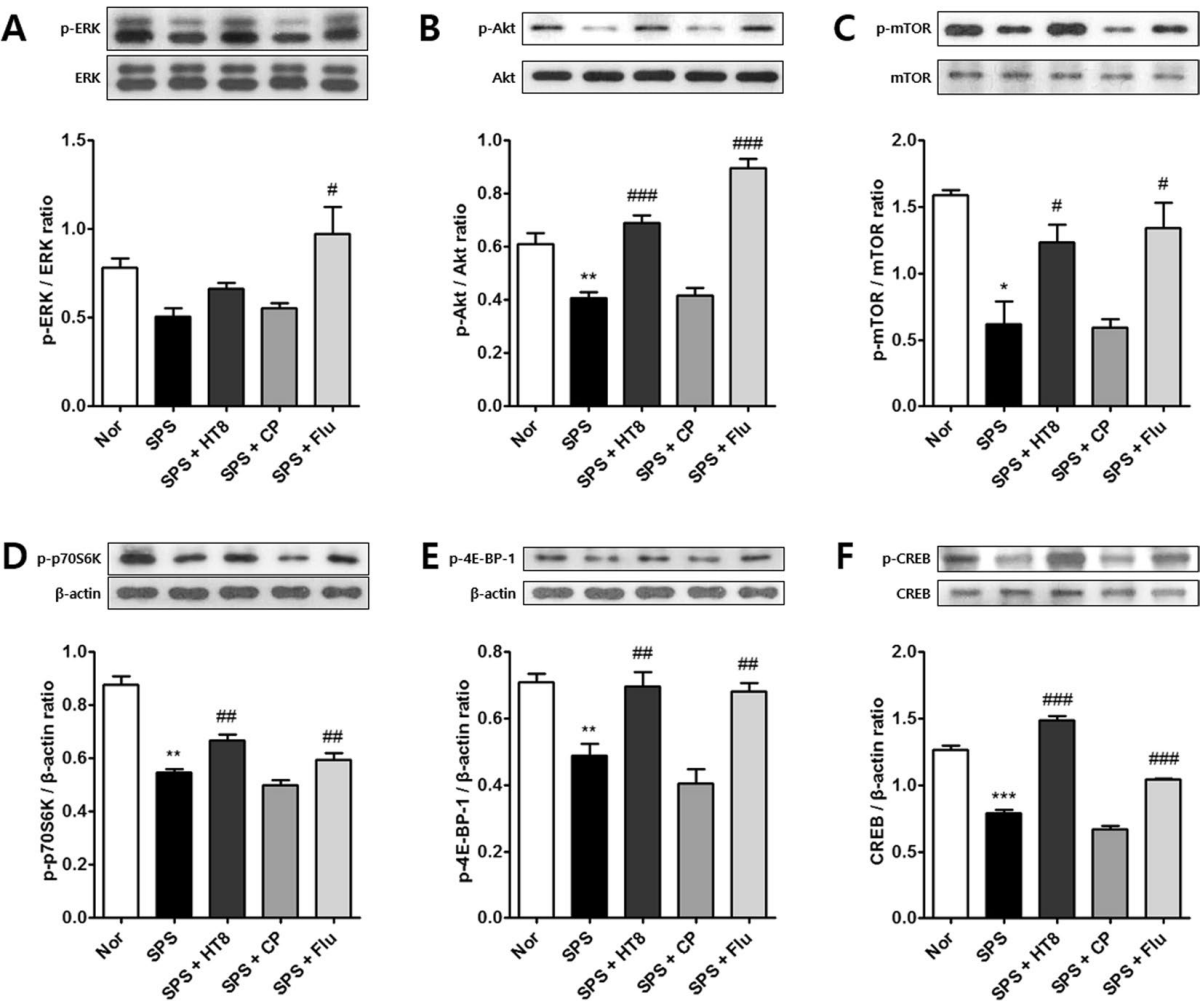
Activation of mTOR signaling pathway in the hippocampus after acupuncture stimulation at HT8. Western blot analysis of protein expression levels of (**A**) ERK, (**B**) Akt, (**C**) mTOR, (**D**) p70S6K, (**E**) 4E-BP-1 and (**F**) CREB in the hippocampus. SPS group showed a tendency to decrease mTOR signaling pathway protein levels. Acupuncture at HT8 was tendency to increase all protein expression levels in the hippocampus. **p < 0.01, *p < 0.05 vs. Nor group, ^###^*p* < 0.001, ^#^*p* < 0.05 vs. SPS group. Nor, normal; SPS, single prolonged stress; HT8, acupuncture group with the HT8; CP, control point acupunctured group; Flu, fluoxetine.

*Post hoc* analysis showed that the levels of, p-ERK (*p* < 0.05, Fig. 4A), p-Akt (*p* < 0.01, Fig. 4B), p-mTOR (*p* < 0.001, Fig. 4C), p-p70S6K (*p* < 0.01, Fig. 4D), p-4E-BP-1 (*p* < 0.01, Fig. 4E), and p-CREB (*p* < 0.001, Fig. 4F) in the SPS group were significantly decreased compared with those in the Nor group. Compared with values in the SPS group, the expressions of p-ERK (Fig. 4A), p-Akt (*p* < 0.001, Fig. 4B), p-mTOR (*p* < 0.001, Fig. 4C), p-p70S6K (*p* < 0.01, Fig. 4D), p-4E-BP-1 (*p* < 0.01, Fig. 4E), and p-CREB (*p* < 0.001, Fig. 4F) were increased by acupuncture stimulation at HT8. In addition, the levels of p-ERK (p < 0.01, Fig. 4A), p-Akt (*p* < 0.001, Fig. 4B), p-mTOR (*p* < 0.001, Fig. 4C), p-p70S6K (*p* < 0.01, Fig. 4D), p-4E-BP-1 (*p* < 0.01, Fig. 4E), and p-CREB (*p* < 0.001, Fig. 4F) were significantly increased in the SPS + Flu group compared with the SPS group.

### Pretreatment with the mTOR inhibitor rapamycin blocked the effect of HT8

To confirm the involvement of the mTOR signaling pathway in the effect of acupuncture at HT8, we injected the rats with rapamycin (Rapa, 110 μg/30 μl; intranasal [i.n.]), an inhibitor of the mTOR pathway, and then performed the behavioral tests.

**Figure 5:**
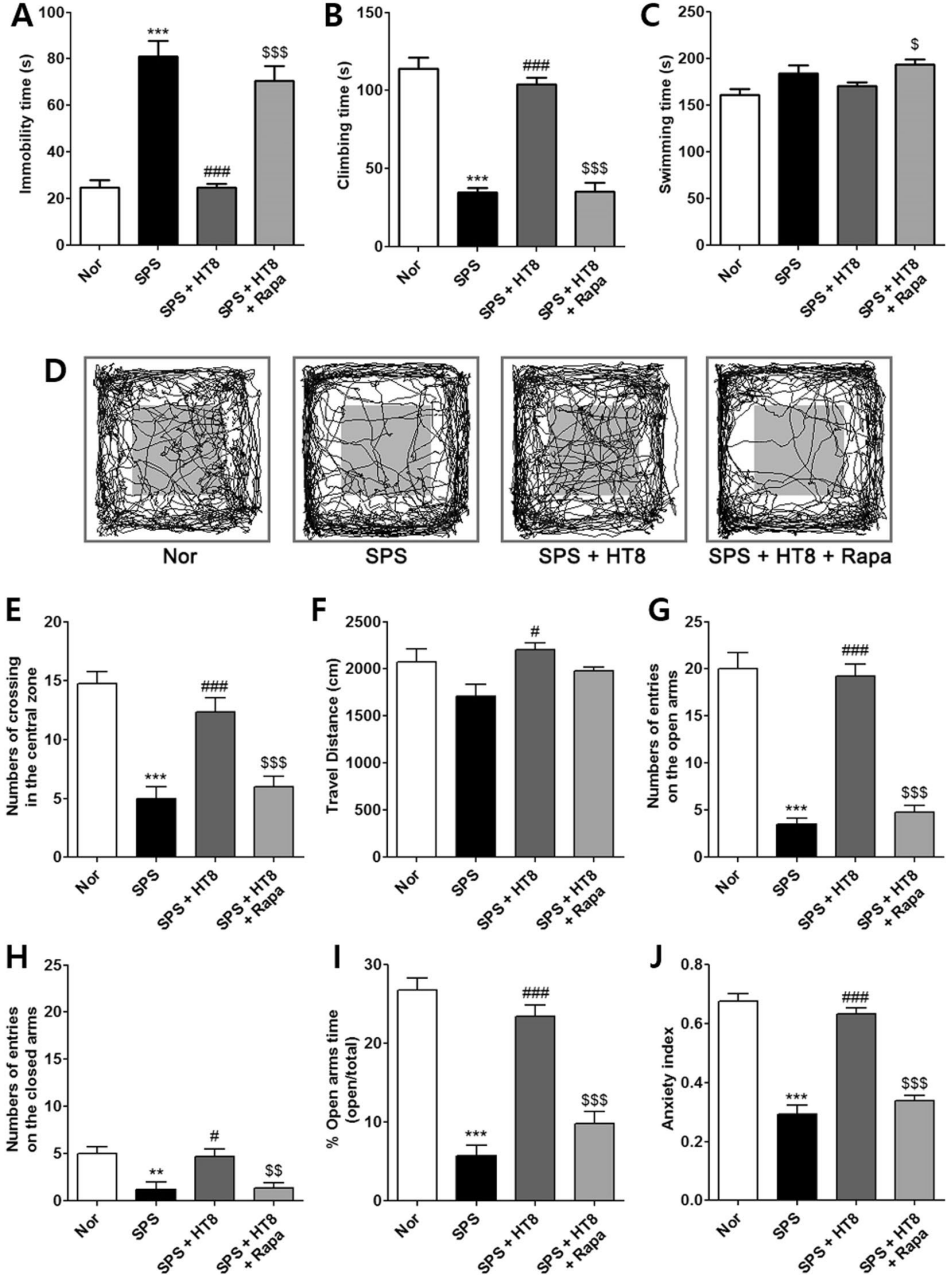
Blocked the antidepressant and anxiolytic effects induced by rapamycin. Effects of acupuncture stimulation at HT8 on depression- and anxiety-like behaviors in (**A**–**C**) FST, (**D**–**F**) OFT, and (**G**–**J**) EPM test in SPS model of PTSD. Pretreatment with mTOR inhibitor, rapamycin (110 μg/30 μl, i.n.), blocked depression- and anxiety- like behavior by acupuncture at HT8 in the hippocampus. ***p < 0.001, **p < 0.01 vs. Nor group, ^###^*p* < 0.001, ^#^*p* < 0.05 vs. SPS group, ^$$$^*p* < 0.001 vs. HT8 group. n = 4–5 each. Nor, normal; SPS, single prolonged stress; HT8, acupuncture group with the HT8; Rapa, rapamycin; FST, forced swim test; OFT, open field test; EPM, elevated plus maze.

One-way ANOVA of the behavior in rats undergoing FST revealed that the SPS + HT8 + Rapa group had significantly lower immobility [F_3,17_ = 33.41, *p* < 0.0001] and climbing time [F_3,17_ = 57.93, *p* < 0.0001]. However, the analysis revealed no significant effect on swimming time [F_3,17_ = 5.218, *p* < 0.0126]. *Post hoc* analyses showed that the SPS + HT8 + Rapa group showed inhibition of antidepressant effects of acupuncture shown in SPS + HT8 group (immobility, *p* < 0.001: climbing time, *p* < 0.001, Fig. 5A,B), but there were no difference in swimming time (Fig. 5C). Taken together, these findings suggest that pretreatment with the mTOR inhibitor rapamycin significantly blocked the effect of HT8 on the depression- and anxiety-like behaviors.

One-way ANOVA indicated that the anxiety-like behavior was significantly blocked in the SPS + HT8 + Rapa group [F_3,13_ = 21.72, *p* = 0.0001]. The effect of acupuncture at HT8 reproduced results of previous experiments. The routes traveled by rats exposed to a novel environment in the OFT indicated that the SPS + HT8 + Rapa group engaged in less exploratory behavior, particularly in the center zone, than did the SPS + HT8 group (Fig. 5D). *Post hoc* analyses showed the SPS + HT8 + Rapa group exhibited fewer line crossings compared to the SPS + HT8 group (*p* < 0.001, Fig. 5E). However, the travel distance did not differ among the groups [F_3,17_ = 3.951, *p* = 0.0311], indicating that their motor function did not differ significantly (Fig. 5F).

The EPM test showed similar results to those of the previous experiments. One-way ANOVA revealed significant effects on the number of entries into the open [F_3,17_ = 53.23, *p* < 0.0001] and closed arms [F_3,17_ = 9.281, *p* = 0.0012] of the maze. According to *post hoc* analyses, the SPS + HT8 + Rapa group showed fewer entries into the open arms (*p* < 0.001, Fig. 5G), but not into the close arms, compared to the SPS + HT8 group (*p* < 0.01, Fig. 5H), indicating that rapamycin inhibited the anxiolytic behavior by acupuncture. Statistical analyses of the EPM data showed that the percentage of open arm time [F_3,17_ = 47.31, *p* < 0.0001] differed significantly among the four groups. *Post hoc* comparisons revealed that the SPS rats spent a significantly lower percentage of time in the open arms of the maze compared to the Nor group (*p* < 0.001, Fig. 5I). However, the rats in the SPS + HT8 group showed a significant restoration of time spent in the open arms compared to that spent by the SPS group (*p* < 0.001). The SPS + HT8 + Rapa group spent a significantly lower percentage of time in the open arms of the maze than did the SPS + HT8 group (*p* < 0.001, Fig. 5I). Overall, one-way ANOVA revealed a significant group effect [F_3,17_ = 66.74, *p* < 0.0001], in the anxiety index calculated based on the number of visits to and time spent in the open and closed arms. SPS + HT8 + Rapa group showed the inhibition of anxiolytic effects of acupuncture (*p* < 0.001; vs. SPS + HT8, Fig. 5J). Therefore, the effect of HT8 treatment on EPM test was significantly blocked by pretreatment with the mTOR inhibitor, rapamycin.

### Effects of acupuncture at HT8 on the expressions of p-mTOR in the CA1, CA3 and dentate gyrus (DG) of the hippocampus using immunofluorescent analysis

**Figure 6:**
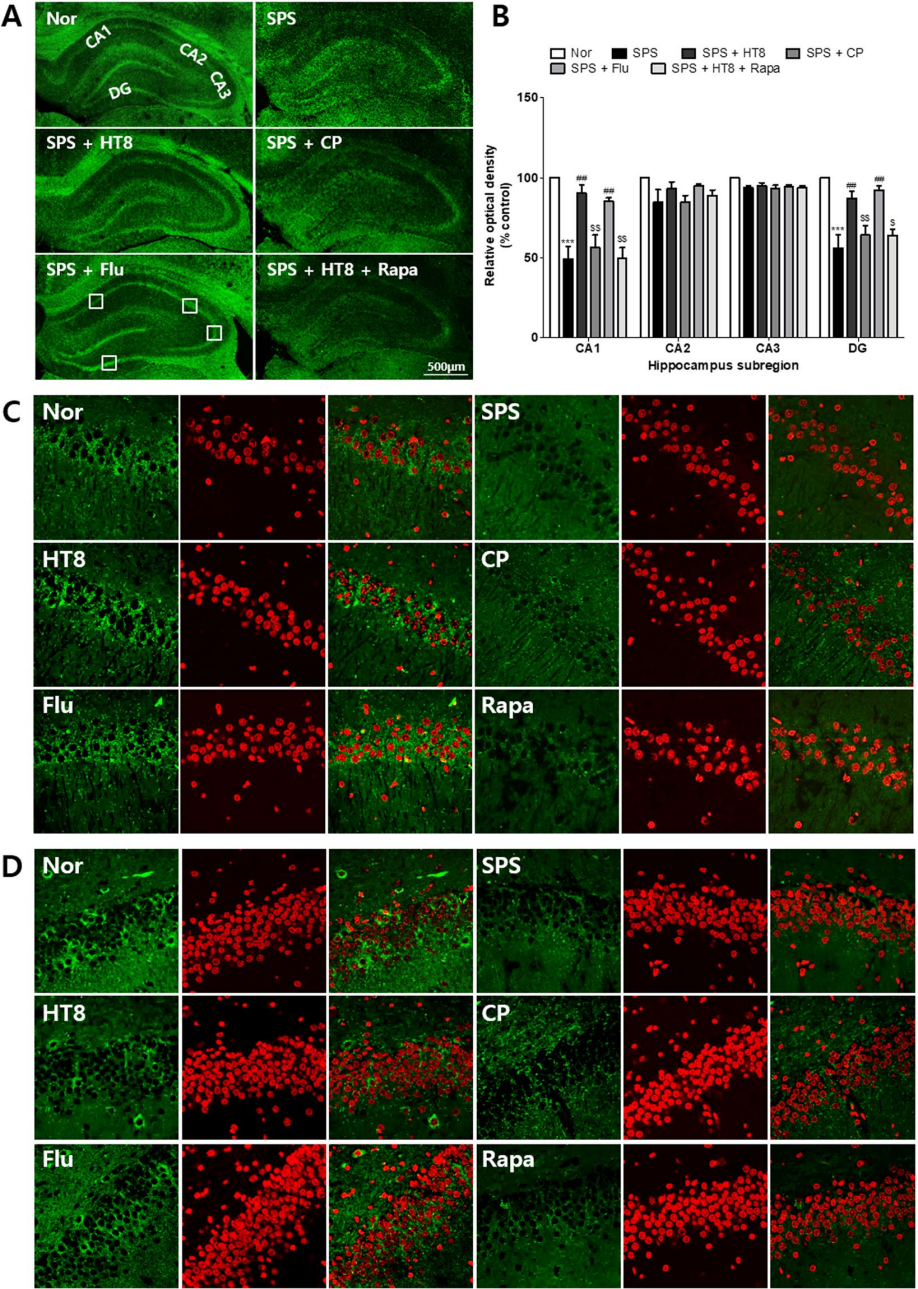
Inhibition of the p-mTOR expression levels of acupuncture by rapamycin in the hippocampus subregion. (**A**,**B**) Immunofluorescence analysis of p-mTOR expression levels in the hippocampus subregion CA1, CA2, CA3 and DG. (**C**) p-mTOR expression levels in the CA1. (**D**) p-mTOR expression levels in the DG. (Cropped blots are displayed; Full-length blots are presented in Supplementary Figure, labeled Fig. 2) ***p < 0.001 vs. Nor group, ^##^*p* < 0.01 vs. SPS group ^$$^*p* < 0.01, ^$^*p* < 0.05 vs. HT8 group. n = 3 each. Nor, normal; SPS, single prolonged stress; HT8, acupuncture group with the HT8; CP, control point acupunctured group; Flu, fluoxetine; Rapa, rapamycin.

We tried to investigate which regions of hippocampus were associated with the changes of p-mTOR by acupuncture, and one-way ANOVA revealed that the densities of p-mTOR were significantly different in the CA1 [F_2,17_ = 15.30, *p* < 0.0001] and DG [F_2,17_ = 13.20, *p* = 0.0002] between the all groups (Fig. 6A,B), while there were no such difference in CA2 [F_2,17_ = 2.063, *p* = 0.1411] and CA3 [F_2,17_ = 3.087, *p* = 0.0509]. *Post hoc* test showed that the level of p-mTOR in SPS group was lower than that in Nor group (*p* < 0.001) in both CA1 and DG. In contrast, those in SPS + HT8 and SPS + Flu groups were significantly higher than SPS group (each *p* < 0.01). The rapamycin injection inhibited these effects of acupuncture (*p* < 0.01; vs. SPS + HT8) (Fig. 6C,D).

### Effects of acupuncture at HT8 on synaptic proteins and brain-derived neurotrophic factor (BDNF) in the hippocampus following SPS

Synaptic plasticity is involved in the pathogenesis of depression. The mTOR signaling pathway is important in regulating protein translation. Thus, we examined the expressions of p-PSD95, p-Syn1, and p-GluR1, the major synaptic proteins related to antidepressant effects and synaptic plasticity. In addition, we investigated changes in BDNF.

**Figure 7:**
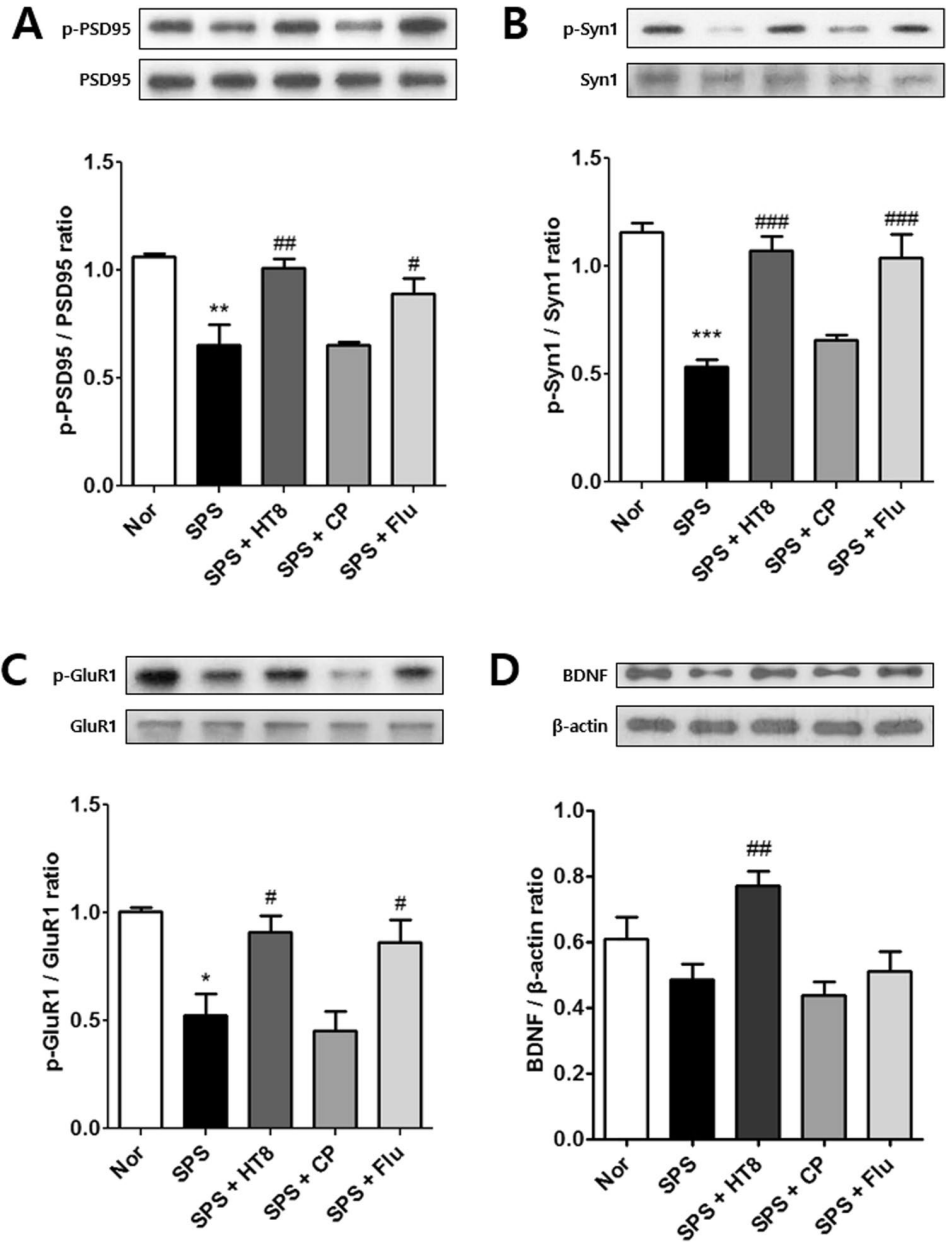
Activation of synaptic proteins in the hippocampus. Western blot analysis of synaptic protein expression levels of (**A**) PSD95, (**B**) Syn1, (**C**) GluR1 and (**D**) BDNF in the hippocampus. SPS group decreased synaptic protein levels. Stimulation of HT8 increased the synaptic protein expression in the hippocampus. (Cropped blots are displayed; Full-length blots are presented in Supplementary Figure, labeled Fig. 3) **p < 0.01, *p < 0.05 vs. Nor group, ^##^*p* < 0.01, ^#^*p* < 0.05 vs. SPS group. n = 3 each. Nor, normal; SPS, single prolonged stress; HT8, acupuncture group with the HT8; CP, control point acupunctured group; Flu, fluoxetine.

Statistical analysis showed significant effects of acupuncture on p-PSD95/PSD95 [F_4,14_ = 11.44, *p* = 0.0009], p-Syn1/Syn1 [F_4,14_ = 4.258, *p* = 0.0288], p-GluR1/GluR1 [F_4,14_ = 8.868, *p* = 0.0025], and BDNF/β-actin [F_4,19_ = 6.354, *p* = 0.0034] protein levels in the hippocampus. A *Post hoc* test revealed that p-PSD95 (*p* < 0.01, Fig. 7A), p-Syn1 (*p* < 0.05, Fig. 7B), and p-GluR1 (*p* < 0.05, Fig. 7C) protein levels were downregulated in the SPS group compared to those in the Nor group. However, the expression levels of p-PSD95 (*p* < 0.01), p-Syn1 (*p* < 0.001), p-GluR1 (*p* < 0.05), and BDNF (*p* < 0.05) were significantly increased in the SPS + HT8 group (Fig. 7A–D). In contrast, the expressions of these synaptic proteins were not significantly affected in the SPS + CP group. The trends in synaptic protein changes in the SPS + Flu group were similar to those in the SPS + HT8 group (Fig. 7).

## Discussion

Our findings show that depression- and anxiety-like behaviors in the SPS rat model of PTSD are alleviated by acupuncture at acupoint HT8, but not at other acupoints. HT8 acupuncture stimulation induces increased mTOR phosphorylation, and upstream and downstream signaling accompanies these behavioral responses. Previous reports suggested that activation of mTOR and subsequent intracellular signaling mediates the behavioral effects of ketamine, a fast-acting antidepressant used clinically. This enhancement of the mTOR signaling pathway increases expression of synaptic formation proteins as well as BDNF. To our knowledge, this study is the first to show the effects of acupuncture treatment on both depression- and anxiety-like behaviors and their underlying mechanism in the SPS-induced PTSD animal model.

To study PTSD symptoms, we employed the SPS-induced rat model. The SPS paradigm is considered to be an experimental model mimicking PTSD because it mirrors most of the neuronal deficits and psychological symptoms seen in patients with PTSD^38^; depression- and anxiety-like behaviors are considered the main symptoms in the SPS-induced PTSD rat model. In this study, we found that SPS administration caused reduced time spent in the center of the OFT and in the open arms of the EPM, as well as increased immobility in the FST, reflecting characteristics of depression and anxiety that are frequent complaints in patients with PTSD^39^.

To determine the optimal acupuncture treatment for PTSD, we first compared the antidepressant and anxiolytic effects of acupuncture at three acupoints; we found that the most effective acupoint in this SPS model of PTSD was acupoint HT8. After acupuncture treatments at HT8, the rats spent more time in the center of the OFT and showed more frequent visits to and increased time spent in the open arms of the EPM. In addition, the acupuncture-treated SPS rats displayed less immobility and increased climbing behavior in the FST, indicating less behavioral despair in these animals than in the SPS group. Acupuncture stimulation at HT7 exerted a significant antidepressant effect on rats tested in the FST, although there was no significant increase in open arm entry in the EPM. We calculated Z-scores to summarize the overall behavioral effects and found that the effect of stimulation at acupoint HT8 was significantly superior to that at acupoint HT7. In contrast, the control acupoint produced no significant effects, indicating that the antidepressant and anxiolytic effects exerted by acupuncture may be point specific. Next, we compared the efficacy of HT8 stimulation with the positive control, fluoxetine, and found that the antidepressant and anxiolytic effects of acupuncture at acupoint HT8 were equivalent to those of fluoxetine, indicating that acupuncture can play a role as a potential antidepressant and anxiolytic therapy.

The hyperactivity of the HPA axis that commonly seen in patients with PTSD^40^, is decreased by clinically effective PTSD drugs. The altering level of stress hormone is one of the significant mechanisms produced by PTSD treatments. Chronic and acute stress activates CRF-immunoreactive neurons in the PVN of the hypothalamus, alters CRF peptide level, and increases serum corticosterone^41,42^. Forced maintenance of high corticosterone levels is associated with the stress response and subsequent depression- and anxiety-like behaviors in patients with PTSD^43^. Here, we observed whether acupuncture modulated the changes of HPA axis caused by PTSD paradigm and found that acupuncture treatment reduced the levels of CRF expression in the PVN and serum corticosterone after acupuncture. Therefore, the modulatory action of acupuncture on the HPA axis could participate in inhibiting stress responses induced by SPS.

The mTOR signaling pathway is highly linked with hippocampal signaling in depression^42,44,45^. mTOR is a serine/threonine kinase that regulates numerous physiological functions of the nervous system^46^. The activation of mTOR is supported by increased phosphorylation of upstream proteins including ERK and Akt, and mTOR then phosphorylates and activates the downstream protein p70S6K, which inhibits eEF2K, thereby decreasing the phosphorylation of eEF2 and effectively inhibiting eukaryotic initiation factor 4E (eIF4E). Phosphorylation of mTOR also hyperphosphorylates the downstream protein 4E-BP-1, thereby decreasing the interaction with elF4E and promote protein translation^47^. Recent findings suggest that synaptic plasticity may play an important role in the neurobiology of depression and effects of antidepressant therapy^48^. In addition, animal studies have demonstrated that changes in synapse type and number after antidepressant treatment in hippocampus^49^. Several studies suggest that the mTOR signaling cascade which regulates protein synthesis plays a role in hippocampal synaptic plasticity^50^. In this regard, the mTOR pathways play a pivotal role in the regulation of translation initiation and protein synthesis in the hippocampus^51^. CREB is a signaling hub in neuronal gene expression^52^ and is also required for the maintenance of LTP^53^, and CREB target genes such as BDNF and TrkB are also crucial to synaptic plasticity^54^. Based on these previous reports, we then observed the protein levels associated with mTOR pathways in the hippocampus to understand how acupuncture modulates these psychological deficits induced by SPS in the hippocampus. As reported for other rodent depression models, we observed that SPS decreased the phosphorylation levels of upstream proteins such as pAkt and pERK as well as mTOR and its downstream p4E-BP-1 and p70S6K in the hippocampus. It also altered the levels of pCREB and BDNF. In contrast, acupuncture treatments recovered these decreased levels of mTOR as well as upstream and downstream proteins, which might imply the initiation of translation crucial for plasticity.

To test whether activation of the mTOR pathway is essential for the therapeutic actions of acupuncture, we tested whether the mTOR inhibitor rapamycin inhibited the acupuncture effects. Interestingly, pretreatment with rapamycin blocked these therapeutic effects induced by acupuncture, whereas the acupuncture group without inhibitor showed the same behavioral effects as shown in the previous experiments. These results confirmed that the antidepressant and anxiolytic effects of acupuncture may be associated with the mTOR signaling pathway.

Next, we investigated which hippocampal regions were involved with these changes. The results showed that the depressive-like behavior induced by SPS is associated with decreased levels of p-mTOR expression in the CA1 and DG, while changes in p-mTOR in the CA2 and CA3 regions were relatively minimal. In contrast, acupuncture and fluoxetine recovered the levels of p-mTOR in the CA1 and DG. These results may be related to a previous study that suggested the activated p-mTOR in the CA1 and DG can be functionally relevant for LTP maintenance^55^. We also observed the neuronal expression of p-mTOR in the CA1. There was also no evidence of any p-mTOR showing GFAP and Iba-1 immunoreactivities, but p-mTOR cells showed NeuN immunoreactivity (Supplementary Fig. 1).

In patients with major depressive disorder, synaptic failure reduces the levels of key proteins including synaptic proteins and AMPA receptor^56^, which is required for hippocampal LTP^57^. Activation of the mTOR pathway results in production of synaptic proteins such as PSD95, Syn1 as well as GluR1^58^. This production increases the density and synaptogenesis of dendritic spines in the hippocampus, leading to antidepressant-like effects in rodents^59^. mTOR activation mainly controls protein translation and activation; thus, local synaptic protein synthesis at the synapse may be associated with the antidepressant and anxiolytic effects of acupuncture^60^. Thus, as a next step, we examined the activation of synaptic proteins related to synaptic plasticity to investigate how changes in the mTOR signaling pathway can contribute to the behavioral improvement. Our results showed that protein activation of PSD95, Syn1, and GluR1 was reduced in the hippocampus by SPS, and acupuncture recovered these reductions significantly. Overall, our results indicate that acupuncture treatment can rescue SPS-induced behavioral deficits by activating the mTOR signaling pathway to promote synaptic protein synthesis and possibly enhance synaptic plasticity. However, to confirm whether acupuncture promotes synaptic plasticity, further studies that observe the dendritic spine or perform electrophysiological recordings of LTP are required^61–63^.

Several hypotheses have been proposed regarding the link between acupoint stimulation and central modulation. Previously, we suggested that local molecular signaling, such as ERK signaling, in the skin layer elicited by peripheral acupuncture stimulation may be linked with central modulation^64^. In addition, the ulnar nerve innervated around acupoints HT8 and HT7 may be associated with effects revealed in an addiction model^65^. However, further investigation is required to define the mechanism linking acupuncture stimulation at HT8 and central modulation in this SPS model.

**Figure 8:**
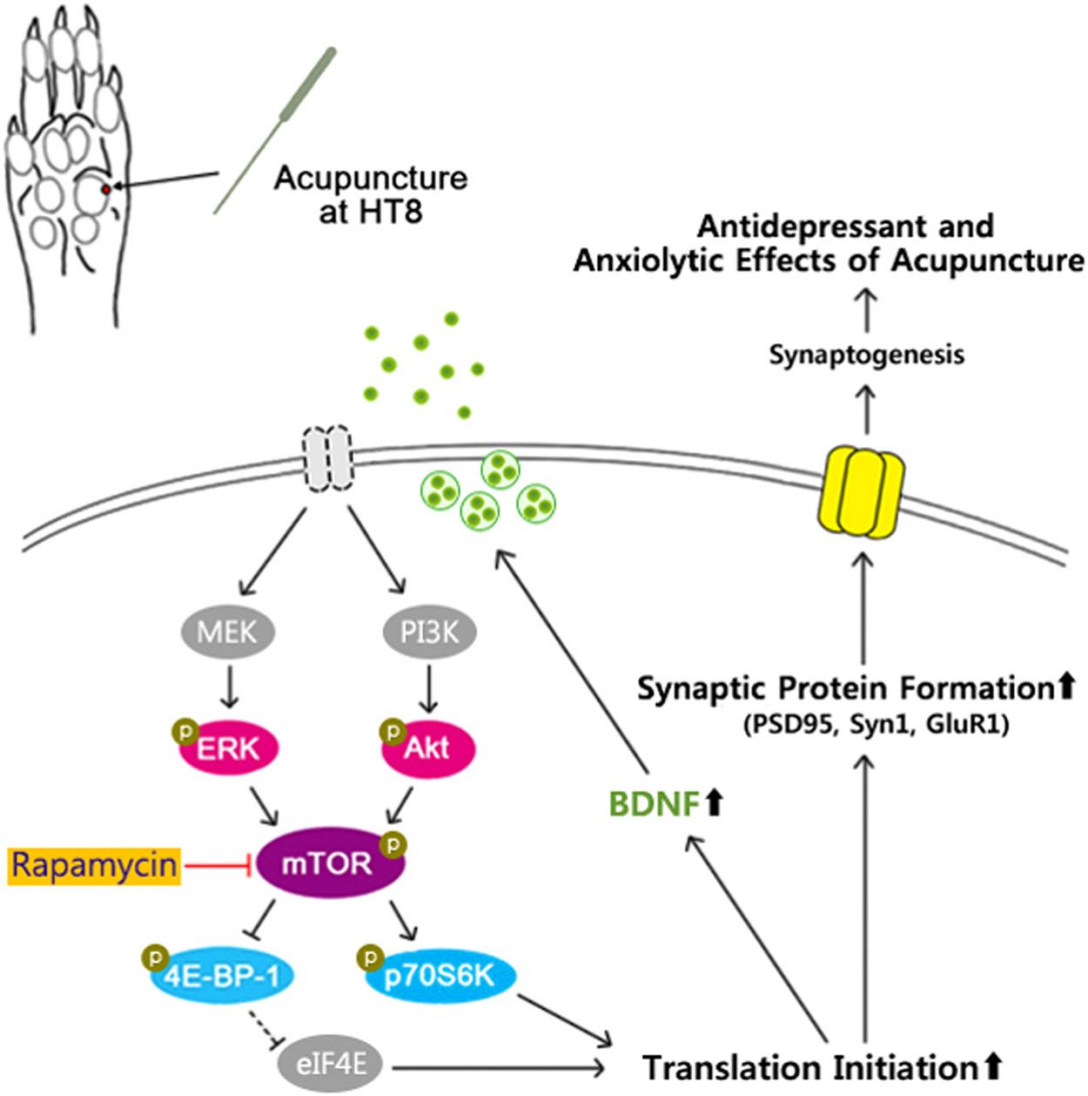
Schematic diagram of the antidepressant and anxiolytic mechanism of acupuncture in a PTSD rat model. Acupuncture stimulation at HT8 release MEK-ERK and PI3K-Akt. Both pathways activate mTOR through phosphorylation. mTOR then phosphorylates and activates p70S6K. In parallel, mTOR hyperphosphorylates 4E-BP-1, reducing its interaction with eIF4E. Together, released of eIF4E from 4E-BP-1 disinhibit protein translation, producing more synaptic proteins such as PSD95, Syn1 and GluR1, as well as BDNF. This facilitates increased synaptogenesis in the hippocampus, and leads to antidepressant and anxiolytic behavior in rodents. However, pretreatment with the mTOR inhibitor rapamycin prevented the antidepressant and anxiolytic effects of acupuncture at HT8. Lines with arrow indicate stimulus connections. Lines with a flattened ends indicates inhibitory connection. The dashed lines indicate the pathways where activity decreased as a result of acupuncture stimulation.

Taken together, acupuncture treatment ameliorated PTSD symptoms elicited by SPS-induced traumatic stress in a rat model. We showed that acupuncture treatment recovered not only behavioral impairment but also changes in serum corticosterone and neuronal CRF, which result from abnormal activation of the HPA axis by traumatic stress. Moreover, we found that acupuncture treatment alleviated the decreased levels of hippocampal mTOR-related signaling proteins, including BDNF and phosphorylated Akt, ERK, p70S6K, and 4E-BP-1. Behavioral enhancement by acupuncture treatment was blocked when pretreated with the mTOR inhibitor, rapamycin. In addition, acupuncture promoted the recovery of hippocampal synaptic proteins, PSD95, Syn1 and GluR1 (Fig. 8). These results suggest that acupuncture treatment alleviates PTSD symptoms via the mTOR signaling pathway and could be a potential candidate for PTSD therapeutics.

## Methods

### Animals

Eight weeks-old adult male Sprague-Dawley (SD) rats (Samtako Animal Co., Seoul, Korea), weighing 200–220 g were used in all the experiments. The rats were maintained on a 12 h light/dark cycle (lights on at 8:00 am, lights off at 8:00 pm.) under controlled temperature at 22 ± 2 °C and the relative humidity at 55 ± 15%. All animals were adapted in this condition for 7 days after they arrived. All animal experiments in this study followed the ‘Guide for Animal Experiments’ edited by Korean Academy of Medical Sciences and were approved by the Institutional Animal Ethical Committee, Dongguk University, South Korea (Permit Number: IACUC-2017-021-1).

### Experimental schedule and group allocation

This study consists of three experiments. First, we examined which acupoints would be suitable for SPS model of PTSD. Second, we compared the effects of selected acupoint and the positive control, fluoxetine, and examined the mechanism associated with the mTOR pathway. Finally, we confirmed that the mediation of mTOR pathway in the acupuncture effects using the mTOR inhibitor, rapamycin.

*Experiment 1*. Rats were randomly divided into five groups:

Normal group (Nor, n = 6),

Single prolonged stress (SPS) group (n = 6),

SPS + Acupuncture at HT7 group (SPS + HT7, n = 6),

SPS + Acupuncture at HT8 group (SPS + HT8, n = 6),

SPS + Control point (SPS + CP) group (n = 6).

*Experiment 2*. Rats were randomly divided into five groups:

Nor group (n = 6), SPS group (n = 6),

SPS + HT8 group (n = 6),

SPS + CP group (n = 6),

SPS + Positive control (Fluoxetine, SPS + Flu) group (n = 6).

*Experiment 3*. Rats were randomly divided into four groups:

Nor group (n = 5), SPS group (n = 5),

SPS + HT8 group (n = 5),

SPS + HT8 + rapamycin group (SPS + HT8 + Rapa, n = 5).

### Single prolonged stress (SPS)

The limbs of the rats were taped with a surgical tape on a metal board and thus the motion of the head was restricted. After immobilizing them for 2 h, they were immediately forced to swim for 20 min in plexiglass cylinder (50 cm height, 24 cm diameter), which was filled two-thirds with 24 °C fresh water. Animals were then dried, allowed to recover for 15 min, and exposed to ether vapor until they lost consciousness. The SPS procedure was performed between 11 am and 4 pm. Afterwards, all animals were individually put into a cage and left undisturbed for 7 days^66^ (Fig. 1A).

### Acupuncture treatment

In order to minimize the restraint stress, rats in all groups were mildly immobilized by holding their necks, making the head to position at an upright position. Acupuncture needles (15 mm in length, 0.20 mm in diameter; Haeng-lim-seo-weon Acuneedle Co., Seoul, Republic of Korea) were bilaterally inserted to a depth of 3 mm at HT7, HT8 or the control point (Fig. 1B). HT8 is located on the palm of the fore paw, in the depression between the fourth and fifth metacarpal bones, proximal to the fifth metacarpophalangeal joint^67^. The angle of needle insertion was 45° to the surface of the skin at HT8 toward the bases of 4th and 5th metacarpal bones (this direction is designed to sedate “*Fire qi*” according to *Sa-Am* acupuncture theory, with our interpretation of reducing anxiety). In the case of HT8, a needle insertion was intended to pass sideways of the common palmar digital nerve of ulnar nerve and common palmar digital artery, which are located between the 4th and 5th metacarpal bones, without directly penetrating the nerve or artery but with stimulating them through rotating manipulation. Also, acupuncture at HT8 was practiced deeply enough to stimulate 4th dorsal interossei muscle beneath the 4th lumbrical muscle. HT7 is located on the anteromedial aspect of the wrist, radial to the flexor carpi ulnaris tendon, on the palmar wrist crease^68^. We also used the “CP” to control the non-specific effects of acupuncture stimulation. CP is located 2 cm to the lateral side of the tail on the gluteus muscle. The needle was inserted perpendicular to the surface of the skin at HT7 and the control point. These inserted needles were turned at a rate of two spins per second for 30 seconds, and removed immediately. The detailed acupuncture technique was shown using ACUSENSOR 2 (Stromatec, Inc., VT, USA, www.stromatec.com) to standardize the acupuncture manipulation used in this study (Fig. 1C–E).

### Pharmacological treatment

As a positive control, Fluoxetine (Flu, 10 mg/kg, Sigma, St. Louis, MO, USA) was applied intraperitoneally (i.p.) after 7 days the exposure to SPS^69^. Fluoxetine was dissolved in a sterile saline solution. It was administered 2 h before the behavioral testing, to maintain the effects during the test period, and to avoid clearance from the central nervous system. The injection was performed on the same day of acupuncture treatment. An mTOR inhibitor, rapamycin (Rapa, 110 μg/30 μl, Cayman, Ann Arbor, Michigan, USA, i.n.) was applied before 30 min stimulated acupuncture. Intranasal administration, a non-invasive method that bypasses the blood-brain barrier, was used to deliver drugs from the nasal cavity into the brain^70^. The rats were held by their ears and 30 μl of the inoculum was gradually released into the nostrils (15 μl in each nostril) with the help of a micropipette.

### Behavior tests

#### Forced swimming test (FST)

In order to evaluate depression severity, we performed a modified FST, according to the previously described method^71^. FST was performed under the same conditions for 5 minutes on day 18 (Fig. 1A). Rats were placed in a cylinder (20 cm diameter × 50 cm height) and filled up to a depth of 30 cm with water at 25 ± 3 °C. Rats were forced to swim for 5 min, and escape behaviors (climbing and swimming) were measured afterward. Immobility behavior represented the time interval in which the rats did not show escape responses. The rats were considered immobile if they were to remain in water without struggling and make only those movements to keep its head above water. Climbing behavior was an upward-directed movement of the forepaws alone the side of the swim chamber. Lastly, movements in the swim chamber, including crossing into another quadrant, defined the swimming behavior.

#### Open field test (OFT)

OFT was used to measure spontaneous locomotor activity of the animals, performed as previously described^72^. OFT test was conducted on day 11 (Fig. 1A). Rats were treated in an open field apparatus in a room. Each of them was individually housed in a rectangular container of dark dim room (60 × 60 × 30 cm). Bottom divided nine identical squares in a room. This material provided best contrast to the white rats in a dimly lit room equipped with a video camera above the center of the room, and their locomotor activities (animal’s movements) were then measured. Locomotor activities were indicated by the speed and the distance of movements, and monitored by a computerized video-tracking system using S-MART program (Pan Lab Co., Barcelona, Spain). The frequency of line crossing and distance traveled in the container was recorded for 5 min.

#### Elevated plus maze (EPM) test

EPM test was used to determine the behavior of approach-avoidance conflict in rats. The test was conducted on day 11 after OFT test (Fig. 1A). As animals explored the maze for 5 min, their behavior was recorded using a video camera located on the ceiling above the center of the maze and this video was relayed to the S-MART program. The following measurements were taken: number of open arm entries, number of closed arm entries, percentage of open arm time, and anxiety index. Arm entry was defined as entering an arm with all four paws. This apparatus consisted of two open arms (50 × 10 cm each) and two closed arms (50 × 10 × 20 cm each) was connected central platform (10 × 10 cm). The maze was raised 50 cm above the floor and made from black Plexiglas. The investigation of the open arms was conducted under indirect dim light. After testing each rat, the maze was cleaned with alcohol. At the beginning of each trial, animals were placed at the center of the maze, facing a closed arm.

Anxiety reduction, as manifested by open arm exploration in EPM test, was defined as an increase in the number of open arms compared to total entries into either open arm or closed arm, and an increase in the percentage of time spent in open arms. Total hours spent on the arms. Total arm entries were also used as an indicator of changes in the locomotor activity of the rats. The anxiety index was calculated as follows:

$${\rm{Anxiety}}\,{\rm{index}}=1-[\frac{(\frac{{\rm{Time}}\,{\rm{spent}}\,{\rm{in}}\,{\rm{the}}\,{\rm{open}}\,{\rm{arms}}}{{\rm{Total}}\,{\rm{time}}\,{\rm{on}}\,{\rm{the}}\,{\rm{maze}}})+(\frac{{\rm{Number}}\,{\rm{of}}\,{\rm{entries}}\,{\rm{to}}\,{\rm{the}}\,{\rm{open}}\,{\rm{arms}}}{{\rm{Total}}\,{\rm{explration}}\,{\rm{on}}\,{\rm{the}}\,{\rm{maze}}})}{2}]$$

Anxiety index values range from 0 to 1 where an increase in the index expresses increased anxiety-like behavior^43^.

### Immunofluorescence

Two hours after FST, the rats were sacrificed with an overdose of entobal and perfused with PBS followed by 10% formalin. The brains were removed, post-fixed overnight, and cryoprotected followed by incubation in 10%, 20%, and 30% sucrose, respectively, for 12 h. The brains were cut into 30 μm coronal sections comprising the PVN of hypothalamus, and selected three sections per rat. Nonspecific binding sites were blocked using 0.3% BSA with 0.3% Triton X-100 in PBS for 1 h at room temperature. After blocking, the sections were incubated with CRF (rabbit polyclonal IgG; 1:500 dilution), p-mTOR (rabbit monoclonal IgG; 1:100 dilution), NeuN (mouse monoclonal IgG; 1:500 dilution), GFAP (mouse monoclonal IgG; 1:500 dilution) and Iba-1 (mouse monoclonal IgG; 1:500 dilution) for 24 h for 72 h at 4 °C. The sections were then incubated in secondary antibody of Alexa Fluor 488 donkey anti-rabbit IgG (A21206, 1:500 dilution) and Alexa Fluor 594 goat anti-mouse IgG (A21206, 1:500 dilution) diluted in PBS with 0.3% Triton X-100 for 1 h at room temperature. The sections were fluorescence mounting medium with DAPI was finally added. The sections were observed under an Olympus FV-1000 laser confocal scanning microscope (Olympus Corporation, Shinjuku, Tokyo, Japan) equipped with a plan apochromatic × 60 oil immersion objective.

### Enzyme-linked immunosorbent assay (ELISA)

The cardiac blood 4 mL was collected two hours after acupuncture treatment of fluoxetine injection and serum was extracted for ELISA. The obtained sample was centrifuged (10,000 rpm for 3 min) and serum was collected and then stored at −20 °C until the assay. Level of corticosterone in the serum was analyzed by ELISA kits according to the manufacturer’s instructions (Enzo Corticosterone ELISA kit; Enzo). The color generated was measured at 590 nm using a spectrophotometric microtiter plate reader (Molecular Devices Corp., CA, USA).

### Western blotting

Brain samples were homogenized in 200 µl of lysis buffer (CyQUANT; Invitrogen, Eugene, OR, USA) including phosphatase inhibitor cocktail tablets and protease inhibitor cocktail tablets. After homogenization, the samples were centrifuged at 12,000 rpm for 15 minutes at 4 °C and the supernatants were collected. The amount of protein was measured using the BCA assay. For western blot analysis, equal protein concentrations (10 µg of total protein) were separated by a 10% sodium dodecyl sulfate-polyacrylamide gel electrophoresis (SDS-PAGE) and then transferred to a PVDF membrane (Millipore, Billerica, MA, USA). The membrane was blocked in 5% skim milk in Tris buffered saline containing 0.1% Tween-20 (TBS-T) and incubated with the primary antibodies overnight at 4 °C. The primary antibodies: p-Akt (1:1000, Cell Signaling Technology, #4058), total Akt (1:1000, Cell Signaling Technology, #4691), p-ERK1/2 (1:3000, Cell Signaling Technology, #4370), total ERK1/2 (1:3000, Cell Signaling Technology, #9102), p-mTOR (1:500, Cell Signaling Technology, #2971), total mTOR (1:500, Cell Signaling Technology, #2983), p-CREB (1:500, Cell Signaling Technology, #9198), total CREB (1:500, Cell Signaling Technology, #9197), p-4E-BP-1 (1:500, Cell Signaling Technology, #9456), p-p70S6K (1:500, Cell Signaling Technology, #9204), p-Syn1 (1:500, Cell Signaling Technology, #88246), p-GluR1 (1:500, Cell Signaling Technology, #8084), p-PSD95 (1:500, Cell Signaling Technology, #45737), total PSD95 (1:500, Cell Signaling Technology, #3409), BDNF (1:300, Santa Cruz Biotechnology, sc-546), and β-actin (1:30000, Sigma-Aldrich, A1978). Then, the membrane was incubated with the secondary horseradish peroxidase-conjugated goat anti-rabbit antibody (Pierce, Rockford, IL, USA) or mouse (Thermo Scientific; PA1-30355) antibodies. The membrane was visualized using a chemiluminescence kit (Super Signal West Pico; Pierce, Rockford, IL, USA). The signal intensities from the immunoblots were analyzed by densitometry.

### Z-score

*Z*-score [(*X*−mean*X*) × s.d.^−1^] was calculated comprising all behavioral measures (based on mean and s.d. of each measure, in each group) to enable the comparison between different measures that depict depression- and anxiety-like symptoms. A result was significant when *p* < 0.05^73^.

### Statistical analysis

GraphPad Prism 5 software (GraphPad Software Inc., San Diego, CA, USA) was used for the statistical analysis. All the data were expressed as the mean ± standard error of the mean (SEM). Group comparisons were performed by one-way ANOVA followed by the Newman-Keuls *post hoc* test. Analyses for body weight was performed using two-way ANOVA with repeated measures and Bonferroni *post hoc* test for pairwise multiple comparisons. In all of the analyses, the differences were considered statistically significant at *p* < 0.05.

## Electronic supplementary material

Dataset 1
