# Supplementary material for: Acupuncture modulates stress response by the mTOR signaling pathway in a rat post-traumatic stress disorder model
Source: Sci Rep. 2018 Aug 8;8:11864. doi: 10.1038/s41598-018-30337-5 (PMC6082850; doi:10.1038/s41598-018-30337-5)
Supplement: Supplementary file 1 — Dataset 1 [file 41598_2018_30337_MOESM1_ESM.doc]

# Supplementary data for:

# “Acupuncture modulates stress response by the mTOR signaling pathway in a rat post-traumatic stress disorder model”

**by Ju-Young Oh, Yu-Kang Kim, Seung-Nam Kim, Bombi Lee, Jae-Hwan Jang, Sunoh Kwon, Hi-Joon Park**

**
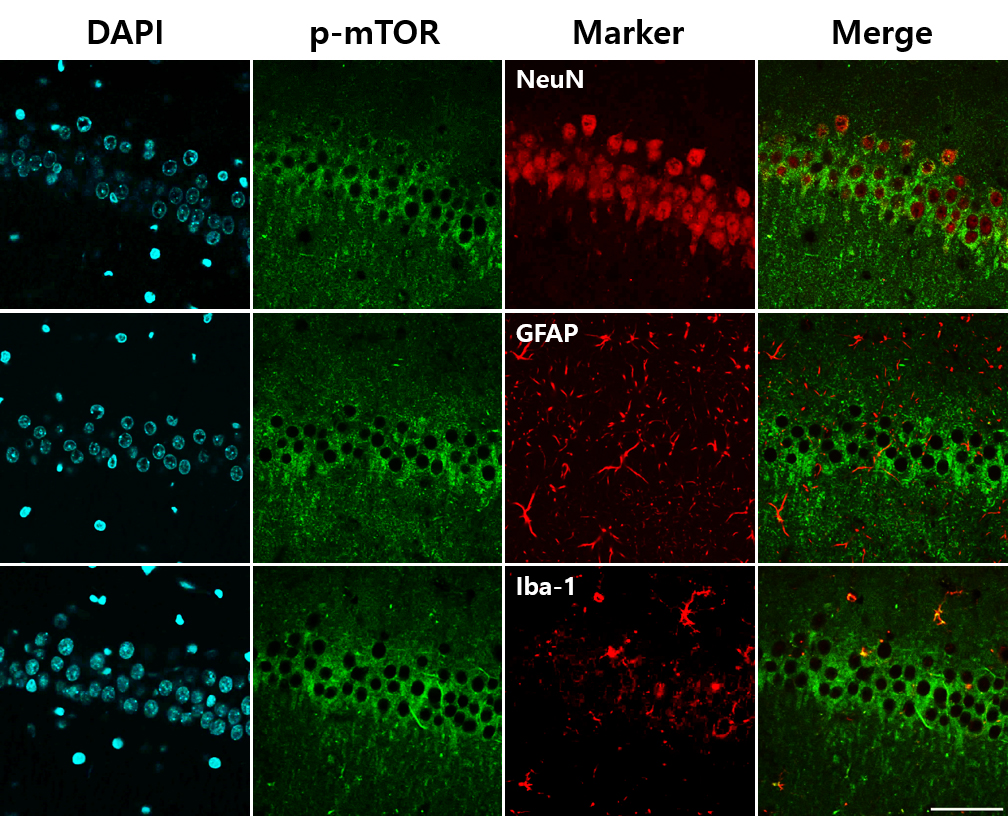
**

**Supplementary figure 1. Double immunofluorescence staining of p-mTOR with NeuN, GFAP and Iba-1.** Double-staining for p-mTOR (green) and NeuN, GFAP or Iba-1 (red) with DAPI (blue) showed p-mTOR immunoreactivity mostly colocalized with a neuronal marker, NeuN, but rarely with an astrocytic marker, GFAP or microglial marker, Iba-1 in the CA1, hippocampus. Scale bar: 50 µm.

**
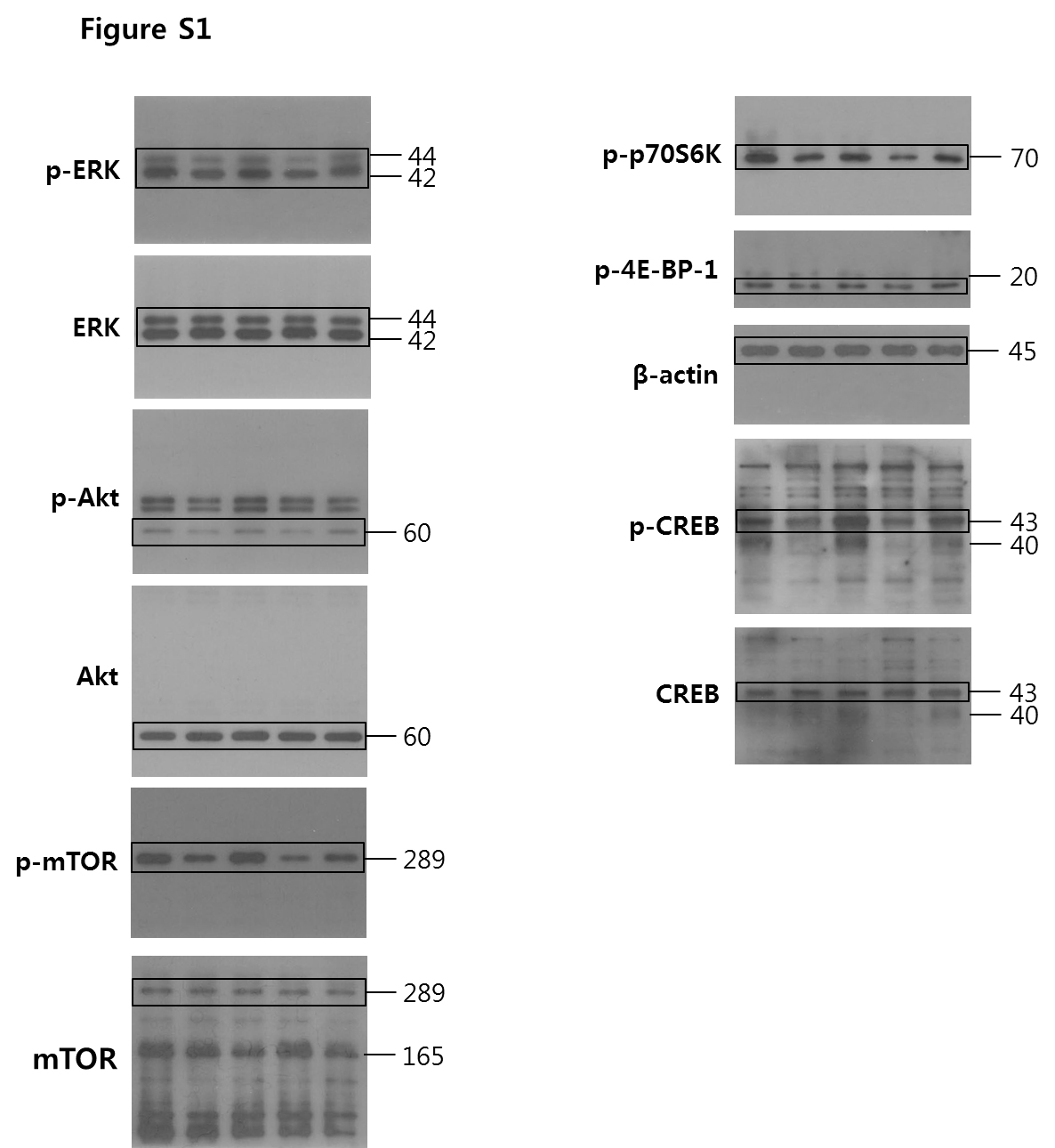
**

**Supplymentary figure 2. Full, original scan of representative western blot image in Figure 4.**

**
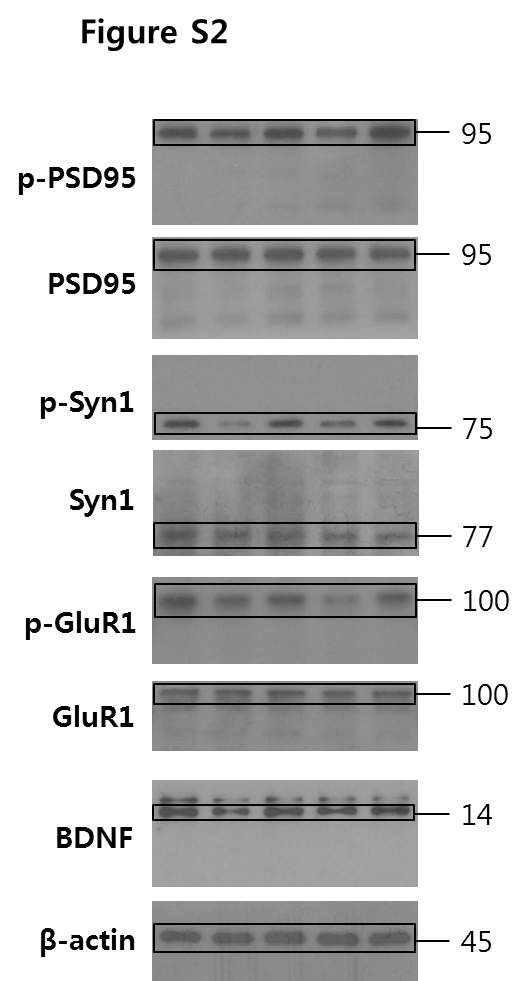
**

**Supplymentary figure 3. Full, original scan of representative western blot image in Figure 6.**
